# Effects of Quorum Sensing Systems on Regulatory T Cells in Catheter-Related* Pseudomonas aeruginosa* Biofilm Infection Rat Models

**DOI:** 10.1155/2016/4012912

**Published:** 2016-03-16

**Authors:** Lei Feng, Qingqing Xiang, Qing Ai, Zhengli Wang, Yunhui Zhang, Qi Lu

**Affiliations:** ^1^Department of Neonatology, Children's Hospital of Chongqing Medical University, Chongqing 400014, China; ^2^Ministry of Education Key Laboratory of Child Development and Disorders, Chongqing 400014, China; ^3^Key Laboratory of Pediatrics in Chongqing, Chongqing 400014, China; ^4^China International Science and Technology Cooperation Base of Child Development and Critical Disorders, Chongqing 400014, China

## Abstract

*Background*. Quorum sensing (QS) systems play an important role in modulating biofilm formation. Recent studies have found that the QS molecules had complex effects on the host immune systems. In addition, regulatory T cells (Tregs), known as important negative regulators in the immune system, have been found upregulated in multiple chronic infections. Therefore, the QS systems were hypothesized to be involved in modulating Tregs in biofilm-associated infections.* Object*. To explore the effects of QS systems on Tregs in catheter-related* Pseudomonas aeruginosa *biofilm infection rat models.* Method*. Catheter-related* Pseudomonas aeruginosa* biofilm infection rat models were established; the bacterial clearance rates, total cell counts in bronchoalveolar lavage (BAL) fluid, pathological changes of lungs, and the levels of Foxp3, TGF-*β*1, and IL-10 in PAO1 strain group were examined and compared with the QS-mutant Δ*lasRΔrhlR* and* ΔlasIΔrhlI* groups.* Results*. In PAO1 group, the bacterial clearance rates were lower, total cell counts were higher, pathological changes were severer, and the levels of Foxp3, TGF-*β*1, and IL-10 were significantly higher compared with QS-mutant groups (*p* < 0.05). No significant difference was observed between the two QS-mutant groups (*p* > 0.05).* Conclusion*. QS systems can trigger host immune system, accompanied with the upregulation of Tregs.

## 1. Introduction 

Cystic fibrosis (CF) is a congenital, recessively inherited disorder, associated with decreased lung function, aggravated pulmonary symptoms, and prolonged duration [1]. The airways of patients with CF are chronically colonized with diverse bacterial, fungal, and viral taxa. Among these microorganisms,* Pseudomonas aeruginosa* (*P. aeruginosa*) are the most commonly isolated organisms [2]. Moreover, in the patients with ventilator associated pneumonia (VAP),* P. aeruginosa* are also the common pathogens [3]. Biofilm, known as the second most popular form of microbes' life, is very beneficial for the* P. aeruginosa* [4], which provides tolerance to the inflammatory defense mechanism. Therefore, antibiotics were found to be less effective at clearing a* P. aeruginosa* biofilm infection compared with a planktonic infection [5].

Quorum sensing (QS) is a cell density-based intercellular communication system, which is a fundamental signaling pathway to participate in regulation of the bacterial virulence and biofilm formation [6, 7].* P. aeruginosa* has three QS systems [8]:* las* system*, rhl* system [9], and quinolone-based system (PQS) [10].* Las* system produces N-3-oxododecanoyl-L-homoserine lactone (3O-C12-HSL) and* rhl* system produces N-butyryl-L-homoserine lactone (C4-HSL) [11]. The* las* and* rhl* systems play a decisive role in PQS system [12]. Moreover, QS systems can interact with host immune defense responses. Recent progress in quorum sensing research has shown that 3O-C12-HSL and C4-HSL strongly increased effective macrophage phagocytosis capacity in the innate immune system during* P. aeruginosa* infections [13]. 3O-C12-HSL can also act as a strong chemoattractant for human neutrophils and induce their migration to the site of lesions in a dose-dependent manner at the early onset of infection [14]. Other studies also proved that 3O-C12-HSL induced production of the chemokine interleukin- (IL-) 8 in fibroblasts and bronchial epithelial cells in human lung [15]. Therefore, QS systems can not only regulate biofilm formation but also interact with eukaryotic cells and modulate host immune response.

In* P. aeruginosa* biofilm-associated infection, the host immune system is characterized by immune-suppressive conditions. It has been confirmed that regulatory T cells (Tregs) could weaken the bacteria clearance and eventually result in a chronic course [16]. It is noteworthy that recent studies have proved that, during* P. aeruginosa* chronic lung infections, lungs developed more Tregs [17]. On the basis of the fact that QS systems modulate both the formation of biofilm and the host immune system and, meanwhile, during biofilm infections the host always shows impaired effector T cells which mainly regulated by Tregs, we hypothesized that QS systems in* P. aeruginosa* could induce Tregs.

## 2. Materials and Methods

### 2.1. Bacteria

PAO1 (wild-type),* ΔlasRΔrhlR* mutant, and* ΔlasIΔrhlI* mutant strains were used in this study (*ΔlasRΔrhlR* and* ΔlasIΔrhlI* strains were kindly provided by Dr. Zhijun Song, Department of Clinical Microbiology, University of Copenhagen, Copenhagen, Denmark). The bacterial strains were cultured in brain-heart infusion (BHI) broth and grown at a neutral pH at 37°C overnight. Overnight-grown cultures of the strains were standardized to 0.05 (OD_600_), and 1 mL contained 2 × 10^7^ CFU × mL^−1^.

### 2.2. Preparation of Endotracheal Tubes Precoated with Bacteria

The tubes for endotracheal intubation, disposable sterile plastic scalp acupuncture tubes of 3.0 mm in diameter, were cut to 1 cm in length, and they were immersed in the above bacteria for 3 days at 37°C. Biofilm was formed on both the inner and outer surface of these inoculation tubes. To better mimic the VAP models, in which the biofilm was mostly formed on the inner surface of the tubes, the outer biofilm was cleared with sterile gauze carefully [18].

### 2.3. Animals

Sprague-Dawley (SD) rats (6–8 weeks of age, 180–220 g) were obtained from the Experimental Animal Center in Chongqing Medical University and housed in a pathogen-free environment in the Laboratory Animal Center at the Children's Hospital of Chongqing Medical University. The experimental protocol was approved by the Animal Care and Use Committee, Chongqing Medical University.

### 2.4. Catheter-Related* P. aeruginosa* Biofilm Infection Models

Twenty-eight female rats were randomly divided into four groups: seven rats of each experimental group were treated with biofilm-covered tubes of PAO1 strain,* ΔlasRΔrhlR* mutant strain, and* ΔlasIΔrhlI* mutant strain, respectively; seven rats in the control group were intubated with sterile tubes. Biofilm-covered tubes were inserted and fixed on the trachea through tracheostomy [19]. The rats were sacrificed at the seventh day after intubation.

### 2.5. Histopathology and Tissue Analysis

Lung tissues were removed from the rats at the time of necropsy, and one-third of the left lung lobe was cut into 1 cm^3^ fixed in 10% neutral buffered formalin for 48 h, embedded in paraffin, cut into 5 *μ*m thick slices, and stained with hematoxylin-eosin (H&E) using standard techniques [20]. Images, at least ten pictures, of one group were obtained using light microscopy (Nikon Eclipse 55i, Japan).

### 2.6. Colony-Forming Units

One-third of the left lung in each rat was homogenized in 1 mL phosphate buffer saline and cultured quantitatively by serial dilution on LB agar plates overnight at 37°C, and then we counted the colonies on the plates to estimate the number of colony-forming units (CFU) [18, 19].

### 2.7. Quantitative PCR Analysis

Total RNA was extracted from one-third of the left lung lobe in each rat. All RNAs were reversely transcribed into cDNA with the PrimeScript RT Regent Kit (Takara, Tokyo, Japan). SYBR Green dye kit (Tiangen, Beijing, China) was used for quantitative PCR analysis. The expressions of GAPDH and Foxp3 at mRNA levels were analyzed. GAPDH was used as the endogenous housekeeping gene to normalize the input mRNA levels. Primers were as follows: Foxp3, forward primer, TCA CTG GCT TTC TGC GTA TGT CC; reverse primer, AGT GCC TGA TGT GCC TGC GAC CT; GAPDH, forward primer, CCT GGA GAA ACC TGC CAA G; reverse primer, CAC AGG AGA CAA CCT GGT CC.

### 2.8. Immunohistochemistry Analysis for TGF-*β*1

Lung tissues were washed, deparaffinized, rehydrated, and subjected to antigen retrieval by boiling in sodium citrate. Immunolabeling was performed using rat anti-TGF-*β*1 (Zhongshanjinqiao, Beijing, China), followed by rabbit anti-rat antibody (Zhongshanjinqiao, Beijing, China). Sections were stained by DAB dyes and then stained by hematoxylin. Image-Pro Plus analysis system was used for semiquantitative statistical analysis.

### 2.9. ELISA for IL-10

After the rats were sacrificed, bronchoalveolar lavage (BAL) fluid was collected by immediate washing of the right lung airway with 3 mL PBS. This fluid was instilled and withdrawn three times [21]. Quantitative assays for protein levels of IL-10 in the BAL fluid were performed using enzyme-linked immunosorbent assay kits (Beijing 4A Biotech Corporation, Beijing, China) according to the manufacturer's instructions. Results were expressed as the mean concentrations in triple BAL fluids.

### 2.10. Statistical Analysis

All the data were statistically analyzed by one-way ANOVA. Differences were considered statistically significant when *p* < 0.05.

## 3. Results

### 3.1. Colony-Forming Units of Bacteria in Lung Tissues

**Figure 1:**
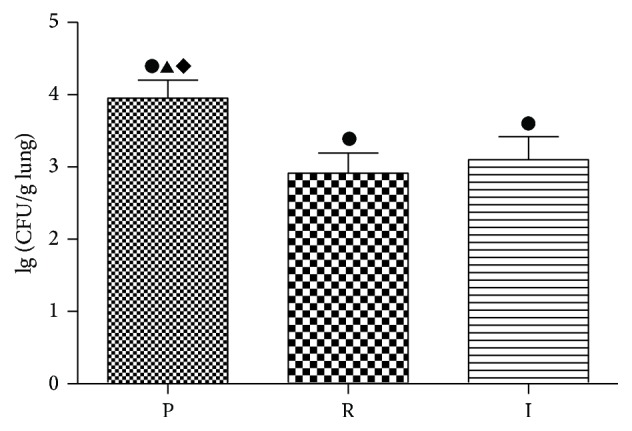
Logarithm of colony-forming units of the three experimental groups. Logarithm of colony-forming units of PAO1 group (P),* ΔlasRΔrhlR* group (R), and* ΔlasIΔrhlI* group (I) in lung tissues after the rats were sacrificed at the seventh day. ●: compared with control group, *p* < 0.05. ▲: statistically significant differences between PAO1 and* ΔlasRΔrhlR* group, *p* < 0.05. ◆: statistically significant differences between PAO1 and* ΔlasIΔrhlI* group, *p* < 0.05.

To evaluate the bacterial clearance of different strains in the rat models, colony-forming units in lung tissues were counted after the rats were sacrificed (Figure 1). Bacterial counts of the lung tissues were determined by plate-dilution method. Compared with the control group, the bacterial counts of three experimental groups were significantly higher. It suggested that the catheter-related* P. aeruginosa* biofilm infection models were successfully established. Two QS-mutant* ΔlasRΔrhlR* and* ΔlasIΔrhlI* groups were significantly lower compared with PAO1 strain (*p* < 0.05), indicating that the bacteria were significantly eradicated when QS systems were absent. However, there was no significant difference between the two QS-mutant strains (*p* > 0.05).

### 3.2. Pathological Morphology of the Rat Lung Infected with Catheter-Related* P. aeruginosa* Biofilm Infection

**Figure 2:**
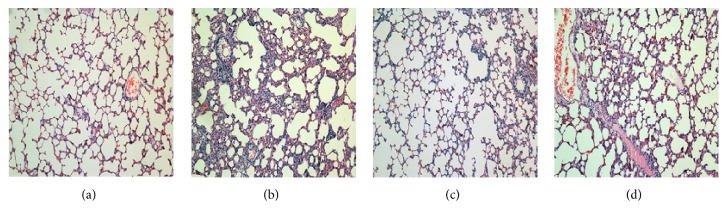
Histopathological changes in the lung of rats. On the seventh day of infection with biofilm-covered tube intubation: control group (a), PAO1 group (b),* ΔlasIΔrhlI* group (c), and* ΔlasRΔrhlR* group (d). H&E stain, original magnification, ×100.

There was no obvious change in the control group. By contrast, the inflammatory cells infiltrated around small- and medium-sized trachea and blood vessels were improved significantly in PAO1 group on the seventh day, with more neutrophil recruitment and severer histological injury (Figure 2). Lung tissues showed signs of necrosis, edema, abscess, and a large area of lung consolidation in PAO1 group. However, no necrosis and only small area of lung consolidation were observed in* ΔlasIΔrhlI* group. Moreover, it had less inflammatory infiltration compared with PAO1. The severity of lesions in* ΔlasRΔrhlR* group was similar to those in* ΔlasIΔrhlI* group but still more serious than the control group.

### 3.3. Total and Cell Counts in the Bronchoalveolar Lavage (BAL) Fluid

**Figure 3:**
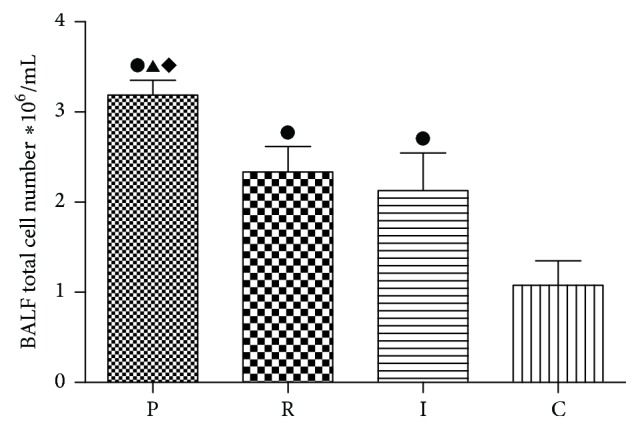
Total cell counts in BAL fluid on the seventh day. PAO1 group (P),* ΔlasIΔrhlI* group (I),* ΔlasRΔrhlR* group (R), and control group (C). ●: compared with control group, *p* < 0.05. ▲: statistically significant differences between PAO1 and* ΔlasRΔrhlR* group, *p* < 0.05. ◆: statistically significant differences between PAO1 and* ΔlasIΔrhlI* group, *p* < 0.05.

To obtain a better understanding of the severity of pulmonary inflammations, total cell counts in BAL fluids were determined (Figure 3). Total BAL fluid cell counts were observed significantly increased in three experimental groups after intubation for seven days (*p* < 0.05). Inflammatory cells in PAO1 group were much higher than those in the two QS-mutant groups (*p* < 0.05).

### 3.4. The mRNA Levels of Foxp3 Were Significantly Enhanced in PAO1 Group Compared with Two QS-Mutant Groups

**Figure 4:**
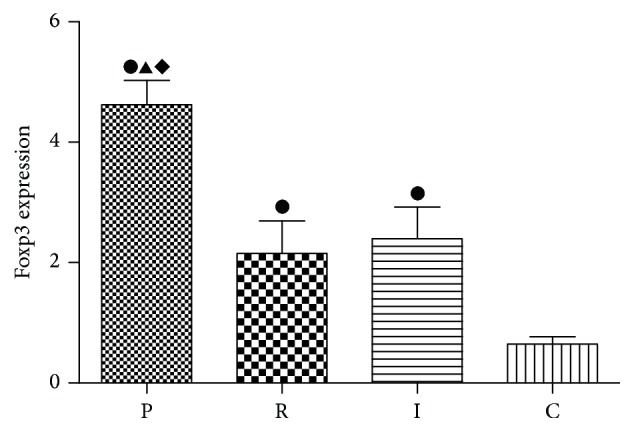
Comparison of mRNA levels of Foxp3 in lung tissues of four groups. PAO1 group (P),* ΔlasIΔrhlI* group (I),* ΔlasRΔrhlR* group (R), and control group (C). ●: compared with control group, *p* < 0.05. ▲: statistically significant differences between PAO1 and* ΔlasRΔrhlR* group, *p* < 0.05. ◆: statistically significant differences between PAO1 and* ΔlasIΔrhlI* group, *p* < 0.05.

It has been confirmed that the transcription factor Foxp3 is considered as a specific marker of Tregs and it could be induced by natural occurring Tregs (nTregs) and induced Tregs (iTregs). Therefore, to quantify the proliferation of Tregs, we identified mRNA levels of Foxp3 (Figure 4). Compared with control group, rats in both PAO1 group and two QS-mutant groups achieved significantly higher mRNA levels of Foxp3 (*p* < 0.05). By contrast,* ΔlasRΔrhlR* and* ΔlasIΔrhlI* groups had lower mRNA levels compared with PAO1 group (*p* < 0.05). However, no significant differences of mRNA levels of Foxp3 between the two mutant groups were observed (*p* > 0.05).

### 3.5. The Treg-Related Cytokines Were Higher in Catheter-Related* P. aeruginosa* Biofilm Infection

**Figure 5:**
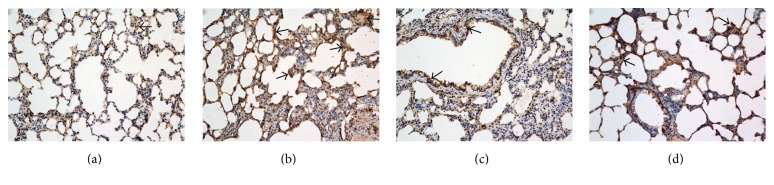
TGF-*β*1 immunohistochemical dyeing in lung tissues. TGF-*β*1 immunohistochemical dyeing in control group (a), PAO1 group (b),* ΔlasIΔrhlI* group (c), and* ΔlasRΔrhlR* group (d). Arrows indicate TGF-*β*1 protein, which was stained yellow and mainly localized in the cytoplasm and cell membrane of positive cells. DAB stain, original magnification, ×200.

**Figure 6:**
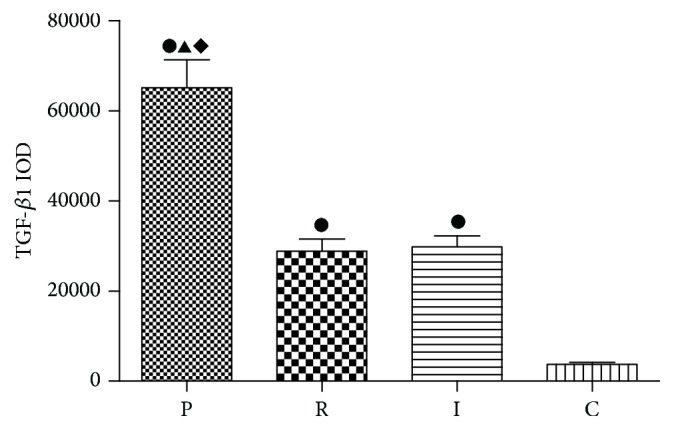
Integrated optical density (IOD) of TGF-*β*1 in lung tissues. Integrated optical density (IOD) of TGF-*β*1 was analyzed by Image-Pro Plus analysis system. PAO1 group (P),* ΔlasIΔrhlI* group (I),* ΔlasRΔrhlR* group (R), and control group (C). ●: compared with control group, *p* < 0.05. ▲: statistically significant differences between PAO1 and* ΔlasRΔrhlR* group, *p* < 0.05. ◆: statistically significant differences between PAO1 and* ΔlasIΔrhlI* group, *p* < 0.05.

It has been confirmed that Tregs could suppress the conventional T cells by releasing immunosuppressive cytokines such as TGF-*β*1 or IL-10 or directly contact with effector T cells or act as antigen presenting cells (APCs). To determine the function of Tregs, we measured the protein level of TGF-*β*1 and IL-10. TGF-*β*1 immunohistochemical dyeing was mainly localized in the cytoplasm and cell membrane of positive cells, with scattered or diffused yellow granular shape (Figure 5). TGF-*β*1 protein staining was high in all three experimental groups. It is noteworthy that a weaker staining of both* ΔlasIΔrhlI* and* ΔlasRΔrhlR* groups was observed compared with PAO1 group. For semiquantitative analysis of these results, integrated optical density (IOD) of each group was analyzed by Image-Pro Plus analysis system (Figure 6).

**Figure 7:**
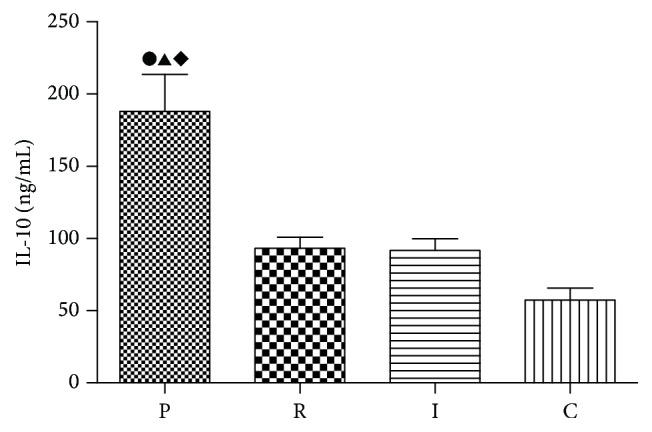
Quantitative assays for protein levels of IL-10 in the BAL fluid. PAO1 group (P),* ΔlasIΔrhlI* group (I),* ΔlasRΔrhlR* group (R), and control group (C). ●: compared with control group, *p* < 0.05. ▲: statistically significant differences between PAO1 and* ΔlasRΔrhlR* group, *p* < 0.05. ◆: statistically significant differences between PAO1 and* ΔlasIΔrhlI* group, *p* < 0.05.

IL-10, another Treg-related cytokine, was also significantly increased in PAO1 group as well as in* ΔlasIΔrhlI* and* ΔlasRΔrhlR* groups compared with the control group. However,* ΔlasRΔrhlR* and* ΔlasIΔrhlI* groups had lower levels of IL-10 compared with PAO1 group (*p* < 0.05). Similar to TGF-*β*1, the concentration of IL-10 had no significant difference between the two QS-mutant groups (Figure 7).

## 4. Discussion

In this study, the relationship between Tregs and* P. aeruginosa* QS systems was investigated in the context of bacterial clearance rates, pathological changes, total cell counts in BAL fluid, and Treg-related cytokines.

Bacterial clearance rates from two QS-mutant* ΔlasRΔrhlR* and* ΔlasIΔrhlI* groups were significantly higher compared with PAO1 group. It has been confirmed that QS systems play an important role in maintaining the structure and function of biofilm [22]. Therefore, the biofilm produced by QS-mutant strains was much easier to be eradicated. This result is consistent with the previous studies which showed that* ΔlasRΔrhlR* and* ΔlasIΔrhlI* strains formed thinner biofilm and the biofilm was more sensitive to the antibiotics [23]. In addition, the observation that bacterial clearance rates from biofilms produced by* ΔlasRΔrhlR* and* ΔlasIΔrhlI* strains were similar suggests that* lasR* and* rhlR* genes are as important as* lasI* and* rhlI* genes in controlling biofilm formation.

In this study compared with PAO1 group, much milder pathological changes in lung tissues of* ΔlasIΔrhlI* and* ΔlasRΔrhlR* mutant groups were indeed observed. Moreover, the pathological changes caused by* ΔlasRΔrhlR* and* ΔlasIΔrhlI* strains had no significant difference. We speculate that* ΔlasIΔrhlI* and* ΔlasRΔrhlR* mutant strains both block* las* and* rhl* signaling pathways, only at two different levels.* P. aeruginosa* possesses two main QS systems (*las* and* rhl*) which drive the production (throughout synthases LasI and RhlI) and the perception (by the transcription factors LasR and RhlR) of the autoinducer signaling molecules 3O-C12-HSL and C4-HSL [24].* ΔlasRΔrhlR* strain was to block the production level.* ΔlasIΔrhlI* strain was to block the perception level. Therefore, the two QS-mutant groups exhibit a significant and similar decrease in the virulence and cytotoxicity [25, 26]. Meanwhile, to obtain a more intuitive understanding of the severity of pulmonary inflammation, total cell counts in BAL fluids were determined. Total cell counts were increased in all three experimental groups compared with the control group. Moreover, compared with two QS-mutant groups, total cell counts were increased more significantly in PAO1 group. These results are consistent with the previous researches which have demonstrated that QS systems could induce multiple human immune cells such as neutrophils, macrophage and aggregate them in the lesions [13, 14].

In this study, the experimental groups all exhibit significant high levels of Foxp3, suggesting that the proliferation of Tregs is promoted. Tregs are mainly involved in the preservation of host homeostasis during infections. Some studies have shown that, in bacterial infections, Tregs usually exhibit immunosuppressive functions by reducing the effector T cells response to infections, which frequently results in cases of chronic disease processes [27]. Similarly, in our study, bacteria were observed to be persisted for seven days without efficient eradication. Therefore, it is possible that, in* P. aeruginosa* biofilm infection, Tregs are activated and their activation counterbalances the immune effectors, eventually leading to a persistent inflammation in airways. In addition, other researchers have found that, during biofilm infection, the immune cells showed frustrated phagocytosis, which is connected with enhanced biofilm resistance [28, 29]. In another animal study using rats infected with agar-entrapped* P. aeruginosa* as the chronic infection models, the researchers also obtained a similar finding [17]. Particularly, a significantly lower mRNA level of Foxp3 was observed in two QS-mutant groups compared with PAO1 group, which indicates that Tregs show a lower level of proliferation when QS systems are absent. Because of the absence of QS systems, Tregs are less activated and therefore the effector T cells are more efficient in eradicating the bacteria. These results provide a good explanation to the observed phenomenon that more QS-mutant strains were cleared during the infection. Moreover, Hector et al. have reported that, in* P. aeruginosa* infection rat models, Tregs were significantly downregulated [30]. The acute lung infection models were established by infecting the rats with planktonic bacteria for 12 h. However, in our study the chronic lung infection models were established by using catheter-related biofilm to infect rats, and the processing time was seven days. We believe these discrepancies may cause the different results.

To further investigate the function of Tregs in* P. aeruginosa* biofilm infection, we mainly identified the protein levels of TGF-*β*1 or IL-10. We found that TGF-*β*1 was much higher in experimental groups. On one hand,* in vitro* studies have proved that, in the presence of TGF-*β* as well as IL-2, conventional CD4+ T cells are able to differentiate into iTregs [31, 32], suggesting that TGF-*β*1 could promote the proliferation of Tregs. On the other hand, TGF-*β*1 is an important immunosuppressive cytokine; the high level of TGF-*β*1 indicates the activation of the suppressive function of Tregs, which is inhibiting the immune defense system [33]. Recent studies found the similar increase in TGF-*β*1 in* P. aeruginosa* chronic lung infections [17]. These results suggest that, in* P. aeruginosa* biofilm-associated infections, Tregs are significantly upregulated and activated. Moreover, QS-mutant strains showed lower levels of TGF-*β*1 and still no significant difference was found between the two QS-mutant groups. We speculate that TGF-*β*1 is mainly secreted by Tregs, so the change of TGF-*β*1 is consistent with Tregs.

The results of IL-10 were similar to TGF-*β*1. IL-10 is another key negative Treg-related cytokine [34]. It has been demonstrated that, in the presence of IL-10, antigen-specific activation of T cells by APCs leads to a type 1 regulatory T cells (Tr1) phenotype [35]. Moreover, IL-10 enhances the differentiation of IL-10-secreting Tregs, and Tregs in turn produce more IL-10, which forms a positive regulatory loop [36]. The function of IL-10 is to inhibit the secretion of IL-2 and to downregulate the expression MHCII. Thereby, IL-10 inhibits the effector T cells. It is possible that, in QS-mutant strains, Tregs are partially activated, which lead to a larger number of activated effector T cells, so that better bacterial clearance and milder inflammation were observed. In conclusion, these results indicate that QS systems participate in upregulating the function of Tregs in biofilm infections.

## 5. Conclusion

It has been demonstrated that quorum sensing systems can modulate the host immune system accompanied with the increase of Tregs during* P. aeruginosa* biofilm infection in our present study. In addition, the activation of Tregs suppresses the immune defense system, resulting in a persistent infection. Therefore, Tregs mediated suppression must be controlled to enable the successful clearance of pathogens. However, we were unable to identify the molecular mechanisms by which* P. aeruginosa*-derived factors regulate Tregs proliferation and function, which requires further immunological and biochemical studies.
